# Genome announcement of sulfate-reducing *Oleidesulfovibrio alaskensis* G20 with novel electron transfer gene annotations

**DOI:** 10.1128/mra.00942-25

**Published:** 2026-03-13

**Authors:** Dheeraj Raya, Vincent Peta, Venkataramana Gadhamshetty, Etienne Z. Gnimpieba, Saurabh Sudha Dhiman

**Affiliations:** 1 Civil and Environmental Engineering, South Dakota Mines 6806 , Rapid City, South Dakota, USA; 2 2DBEST Center, South Dakota Mines 6806 , Rapid City, South Dakota, USA; 3 Biomedical Engineering Department, University of South Dakota 730450, Sioux Falls, South Dakota, USA; 4 Chemistry, Biology, and Health Sciences, South Dakota Mines 6806 , Rapid City, South Dakota, USA; Wellesley College, Wellesley, Massachusetts, USA

**Keywords:** illumina sequencing, sulfate-reducing bacteria, extracellular electron transfer, genomic adaptations, stress response, biofilm formation

## Abstract

*Oleidesulfovibrio alaskensis* G20 is a gram-negative, mesophilic, sulfate-reducing bacterium known for its biofilm-forming and bio-corroding characteristics. A total of 173 new gene annotations have been identified, encoding electron transportation proteins, among others. These genes are likely involved in extracellular electron transfer and interspecies interactions, highlighting microbial adaptations for extreme anaerobic environments.

## ANNOUNCEMENT

*Oleidesulfovibrio alaskensis* G20 (OA-G20) is an anaerobic sulfate-reducing bacterium (SRB) (1, 2), a derivative of *Desulfovibrio desulfuricans* G100A (3), isolated from an oil-producing well (Ventura County, California), and lacking the endogenous cryptic plasmid pBG1 (4). Given SRB’s adaptability in extreme conditions (5, 6) and environmental significance (7), the whole genome of the OA-G20 was re-sequenced in 2023. The original attempt at sequencing was performed in 2011 through the whole-genome shotgun technique (8). We adopted the hybrid sequencing and assembly approach, resulting in the annotation of an additional 173 genes.

OA-G20’s genomic DNA was extracted using the extraction kit (Promega) according to the manufacturer’s protocol and cryopreserved in the laboratory. DNA was extracted from 7-day-old cells growing (pH 7.0; 37°C, 150 rpm) in Lactate C medium (6) and used for both short and long read sequencing. The short-read sequencing was performed using Illumina NovaSeq 6000 paired-end with the xGenTM DNA EZ library prep kit (IDT), resulting in a total of 40.3 million reads, each with a read length of 150 base pairs (bp). Trimmomatic v0.36 (9) default parameters were used for quality trimming. Long-read sequencing was performed using a rapid barcoding kit (SQK-RBK114.24) on a MinION flow cell R10.4.1 device (Oxford Nanopore). All subsequent data analyses were performed using default parameters except where otherwise noted. Base calling was performed with Dorado v7.4.12 using a super-accurate basecalling method with a quality score threshold of 10 and a minimum length of 5,000 bp. Long read sequencing yielded 259 Mb from 35,359 reads with a mean length of 7,326.4 bp and an N_50_ of 7153 bp. The hybrid genome assembly was performed using Unicycler v0.5.1 (10), integrating both short- and long-read data. Reports (11) show that adopting short-read assembly alone may result in ~13% of genes being unaccounted for due to assembly failures, highlighting the advantage of the adopted hybrid approach, which is reproducible and can be applied to other bacterial strains.

Annotation of the hybrid assembly was performed using NCBI Prokaryotic Genome Annotation Pipeline v6.10 (12) and confirmed by Bakta v1.11.0 (13), which identified 3,379 putative genes, 66 tRNAs, 11 rRNAs, 5 ncRNAs, 5 pseudogenes, compared to 3,258 protein-coding genes and 25 pseudogenes previously annotated (CP000112.1) (8). The hybrid approach yielded a genome of 3,732,758 bp with a 57.83% GC content, compared to a reference genome of 3,730,232 bp with 58.84% GC content (8). The assembly achieved a genome fraction of 99.98% compared to the reference genome. BUSCO analysis (14), based on the Desulfovibrionales lineage data set (*n* = 777), confirmed the high completeness of the genome, with 99.6% of BUSCO genes identified.

**Fig 1:**
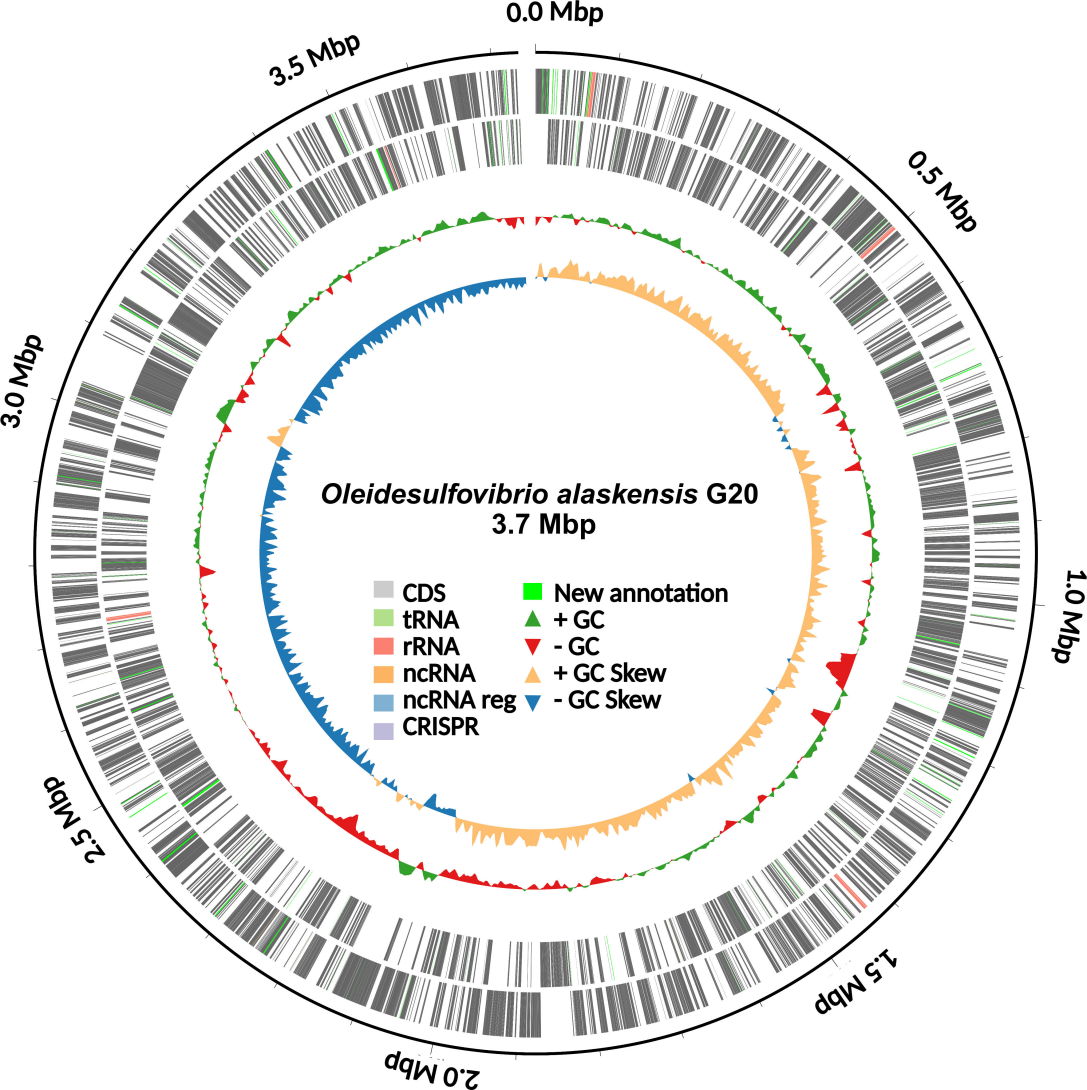
Circular chromosome map of the complete genome of *Oleidesulfovibrio alaskensis* G20. Rings (outermost) illustrate coordinates (Mega basepairs; Mbp), protein-coding genes on forward (outer) and reverse strands, percentage GC content, and GC skew.

The new annotations reveal protein domains (Fig. 1), such as DNA-binding (Integrase/Recombinase), electron transfer (4Fe-4S Ferredoxin), and stress response elements (ribosomal RNA methyltransferase, heat shock proteins), which further validate the stability (15, 16) of OA-G20 genome for extreme environments (17, 18).

## Data Availability

The whole-genome project results are deposited in GenBank as BioProject PRJNA1181794, BioSample SAMN44570664, SRA accession numbers SRR31217235, SRR33254063, and accession number CP187935.
